# Proteomic Analysis on Human Islets Suggests Nucleocytoplasmic Transport as a Mechanism of PERK Attenuation Effects in Diabetes

**DOI:** 10.1016/j.mcpro.2026.101588

**Published:** 2026-05-18

**Authors:** Yeon Soo Park, Soeun Yun, Min Seop Sim, Eun Jin An, Jin Lee, Eun Hee Ha, Jin-Young Jang, Wooil Kwon, Dohyun Han, Hye Seung Jung

**Affiliations:** 1 Division of Endocrinology and Metabolism, Department of Internal Medicine, Seoul National University Hospital, Seoul, Republic of Korea; 2 Proteomics Core Facility, Biomedical Research Institute, Seoul National University Hospital, Seoul, Republic of Korea; 3 Department of Transdisciplinary Medicine, Seoul National University Hospital, Seoul, Republic of Korea; 4 Department of Biomedical Sciences, Seoul National University College of Medicine, Seoul, Republic of Korea; 5 Department of Surgery and Cancer Research Institute, Seoul National University College of Medicine, Seoul, Republic of Korea

**Keywords:** proteomics, PERK inhibitor, glucolipotoxicity, β-cell dysfunction, nucleocytoplasmic transport, PDX1, FOXO1

## Abstract

Partial downregulation of pancreatic endoplasmic reticulum kinase (PERK) activity recovered insulin content in human islets exposed to glucolipotoxicity (GLT), resulting in improved insulin secretion and glucose-lowering effects in a mouse model of type 2 diabetes. We conducted this study to elucidate the beta-cell-enhancing mechanisms of PERK attenuation. Pancreatic islets isolated from non-diabetic living donors were divided into three groups: control, GLT mimicking diabetes conditions, and GLT with treatment of a PERK inhibitor (PERKi, GSK2606414) for 24 h. Proteomic analysis was conducted on these samples. Differentially expressed proteins (DEPs) altered by GLT and reversed by PERKi were analyzed using bioinformatics. Validation studies were followed using western blotting, RT-PCR, and immunocytochemistry. Using nine islet samples pooled from seven participants, 161 DEPs were identified among 5513 quantifiable proteins across the three groups. On a subset of 42 proteins that were downregulated by GLT and upregulated by PERKi, GO, and KEGG analyses highlighted nucleocytoplasmic transport (NCT) as a key pathway, involving genes such as *XPO4, KPNA4, NUP43,* and *NUP58*. The involvement of NCT, particularly *XPO4*, was further supported by a replication proteomic analysis using islets from four independent donors. Based on these findings, we examined the NCT of representative β-cell transcription factors, including PDX1 and FOXO1. PERKi significantly increased their nuclear localization under GLT conditions (both *p* < 0.05), accompanied by heightened expression of their target genes, such as *FBXW5.* These results suggest that PERKi-mediated modulation of NCT may enhance the functional activity of PDX1 and FOXO1. In conclusion, proteomic analysis revealed that PERKi appears to modulate NCT of human islets under metabolic stress, thereby contributing to the restoration of β-cell function through regulation of relevant transcription factors. These findings suggested a novel mechanism of low-dose PERKi as a therapeutic approach to diabetes, in addition to the canonical unfolded protein response.

## Highlights

• PERK inhibitor (PERKi) restores β-cell function under glucolipotoxic stress.
• Proteomics revealed that PERKi partially reverses GLT-induced alterations.
• PERKi-restored proteins were enriched for nucleocytoplasmic transport (NCT).
• PERKi regulated NCT of key β-cell transcription factors in mouse and human islets.
• NCT regulation is a targetable mechanism of PERKi in β-cell metabolic stress.

## Graphical Abstract

**Figure:**
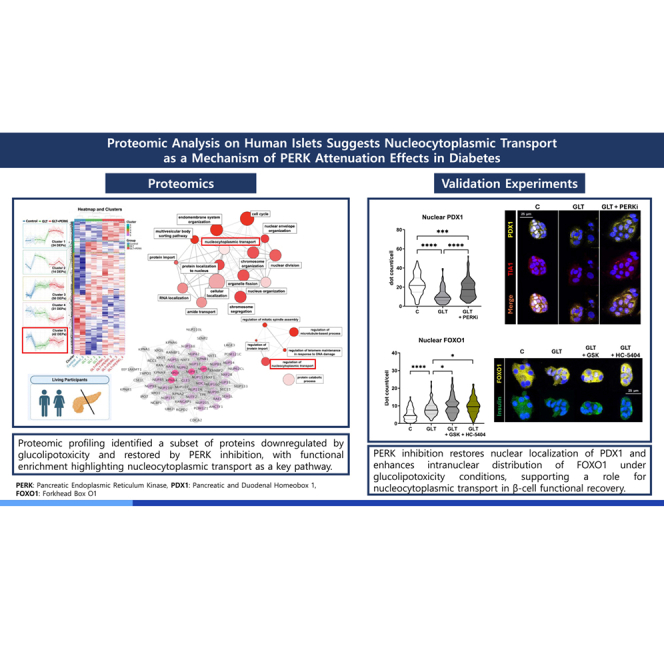

## In Brief

Using proteomic analyses of human islets from living donors, we show that partial inhibition of pancreatic endoplasmic reticulum kinase (PERK) reverses glucolipotoxicity-induced proteomic alterations, particularly restoring proteins involved in nucleo-cytoplasmic transport. This pathway was consistently identified across independent cohorts and further validated by showing that PERK inhibition modulates the nuclear localization of key β-cell transcription factors in a heterogeneous manner. These findings identify nucleo-cytoplasmic transport regulation as a novel therapeutic mechanism of PERK attenuation to preserve β-cell function in diabetes.

Glucolipotoxicity (GLT), resulting from chronic hyperglycemia and elevated free fatty acid levels associated with diabetes, is considered a pathogenic factor contributing to the progressive decline in pancreatic β-cell function and viability (1, 2). Previously, we have reported that partial attenuation of pancreatic endoplasmic reticulum kinase (PERK) activity reversed the decline in human β-cell function induced by GLT (3).

PERK is an ER membrane protein essential for regulating β-cell development, insulin transport, and cell survival. Genetic compromise of *Perk* in mice induced pancreatic atrophy and insulin insufficiency along with diabetes, and *PERK* loss-of-function mutations in humans are associated with neonatal diabetes (4, 5).

In contrast, the effects of partially reducing PERK activity were different. Heterozygous *Perk* deletion in mice increased pancreatic insulin content and insulin secretion, alleviating hyperglycemia in various contexts (6, 7, 8). Specific PERK inhibitors (PERKi), GSK2606414 and GSK2656157, at low doses near their IC_50_, enhanced glucose-stimulated insulin secretion both *in vitro* and *in vivo*, regardless of the presence of metabolic stress mimicking diabetes (9, 10). These findings suggest that targeting PERK signaling could be a promising therapeutic strategy to mitigate β-cell dysfunction in diabetes.

However, the mechanisms by which PERK inhibition affects β-cell function are not yet fully understood. Although PERK is well known to trigger unfolded protein response (UPR) in response to ER stress (11), the effective doses of PERKi appeared to be much lower than those needed to inhibit UPR signaling (9, 12). We have shown that suppressed autophagy was restored by PERKi in an ATG7-dependent manner (3), however, there are many links yet to be revealed. Moreover, glucose-lowering effects of PERKi were also observed in β-cell-specific *Atg7*-deleted mice (9), suggesting PERKi-triggered signaling other than the ATG7-dependent one.

As proteomics is a powerful tool for investigating the molecular mechanisms underlying various diseases and treatments (13), this study aimed a comprehensive proteomic analysis of human islets exposed to GLT with and without PERKi, highlighting the therapeutic potential of PERK attenuation in preserving β-cell function in diabetes.

## Experimental Procedures

### Experimental Design and Statistical Rationale

To investigate the *in vitro* effects of PERKi on pancreatic islets under metabolic stress, this study employed three treatment conditions: control, GLT representing diabetic conditions, and GLT with PERKi. Human islets isolated from non-diabetic living donors were used for these treatment sets; however, in some donors, the control group was omitted because of limited islet yield. Following treatments, islets were cryopreserved until proteomic profiling.

Two independent proteomic approaches were performed using separate donor cohorts. The first was a tandem mass tag (TMT)–based proteomic analysis, which included 2 to 4 experimental replicates and 4 to 7 biological replicates. The variability in replicate numbers reflected the necessity to pool cryopreserved islet samples to obtain sufficient protein. The second approach utilized data-independent acquisition mass spectrometry (DIA-MS), consisting of three experimental replicates and four biological replicates. In this analysis, each donor’s islets were processed as a complete experimental set, while in two cases, samples were combined to ensure adequate protein yield. Given the *in vitro* nature of this study, the numbers of experimental and biological replicates were considered sufficient to detect statistically meaningful differences using one-way ANOVA.

Based on the proteomic findings, selected pathways were further validated using mouse and human islets. Each validation experiment followed the same three-group treatment scheme and included 3 to 5 experimental or biological replicates per condition, which was considered sufficient to detect statistically meaningful differences using one-way ANOVA. For cell-level assays, two biological replicates were assessed across hundreds of technical replicates for each group.

### Participants

Human pancreatic tissues were collected from non-diabetic participants who underwent pancreatic surgery for localized lesions at Seoul National University Hospital (SNUH) between 2021 and 2025. The study was approved by the Institutional Review Board of SNUH (IRB# 2110-151-1266 and 0901-010-267) and conducted in accordance with the principles of the Declaration of Helsinki. All participants provided written informed consent. Criteria for enrollment are presented in Table S1.

### Preparation of Islets

Following surgical resection of the pancreas, a portion of the resection margin consisting of grossly normal-appearing tissue was immediately transported to a clean bench in histidine–tryptophan–ketoglutarate (HTK) solution. Tissues were enzymatically digested with collagenase NB1 (SERVA Electrophoresis GmbH, cat. DS17455.03) and neutral protease NB (SERVA Electrophoresis GmbH, cat. S3030111) and subsequently purified using Ficoll density gradient centrifugation. The islets were then washed with Hanks’ balanced salt solution (HBSS; LB 003-02, WELGENE), hand-picked, and incubated overnight to allow recovery in culture media consisting of CMRL medium (Gibco/CMRL Medium 1066) supplemented with 10% FBS (Gibco/Fetal Bovine Serum), bovine serum albumin (BSA; Sigma-Aldrich/A9647-50G), nicotinamide (Sigma-Aldrich/N0636-100G), and L-glutamine (Sigma-Aldrich/G7513-100 Ml).

**Fig. 1:**
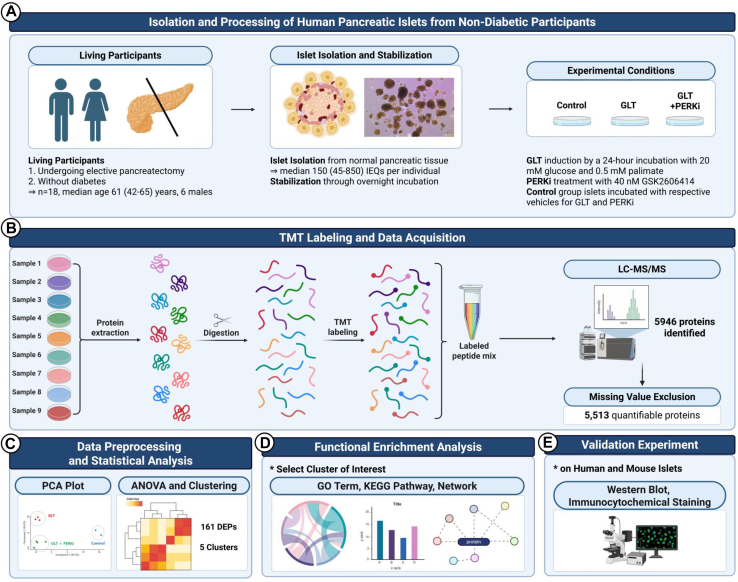
**Overview of the Study.***A*, human islets were isolated from non-diabetic patients undergoing pancreatectomy, and those from each individual were allocated into three treatment conditions: control, GLT, and GLT + PERKi for 24 h. *B*, Nine samples pooled from seven participants were subjected to TMT-based quantitative proteomic analysis, identifying 5513 quantifiable proteins. *C*, statistical analysis revealed 161 DEPs. *D*, functional enrichment analysis was performed using DEPs. *E*, based on bioinformatic analysis, validation experiments were conducted. ANOVA, analysis of variance; DEP, differentially expressed protein; GLT, glucolipotoxicity; GO, gene ontology; IEQ, islet equivalent; KEGG, Kyoto Encyclopedia of Genes and Genomes; LC-MS/MS, liquid chromatography–tandem mass spectrometry; PCA, principal component analysis; PERKi, pancreatic endoplasmic reticulum kinase inhibitor (GSK2606414); TMT, tandem mass tag.

After overnight incubation, islets were allocated into two to three treatment groups depending on the islet yield. Following completion of the 24-h treatments and washing with PBS, islets were snapfrozen in liquid nitrogen on a donor- and group-specific basis and stored at −80 °C until further analysis (Fig. 1*A*). In some validation experiments, mouse islets were also used, isolated from adult C57BL/6 mice using ductal injection of collagenase as described previously (IACUC approval #25-0184-S1A0) (3).

### Treatment: Glucolipotoxicity With/Without PERK Inhibitor

To induce GLT, islets were incubated for 24 h in the culture medium containing 20 mM glucose and 0.5 mM palmitate. Palmitate was conjugated with fatty acid-free BSA at a 3:1 M ratio (Sigma-Aldrich). GSK2606414 (Merck-Millipore KGaA) was used to attenuate PERK activity, with DMSO (Sigma) serving as the vehicle control. The low dose was 40 nM (approximately the IC_50_ for PERK phosphorylation), and the high dose was 400 nM (approximately the IC_50_ for EIF2A phosphorylation) (12). Control group islets were incubated in culture medium containing the respective vehicles for GLT and PERKi. To assess potential class effects of PERK inhibition, an additional PERK inhibitor with a distinct chemical scaffold, HC-5404 (160 nM; MedChemExpress), was examined (14).

### Sample Preparation for TMT-Based Proteomic Analysis

Treated islet samples with limited yield were pooled from two to three donors to generate a single sample to reach at least 100 islet equivalent (IEQ) per sample. Islet samples were lysed in a lysis buffer containing 4% SDS, 0.1 M Tris-HCl (pH 7.5), and 2 mM Tris(2-carboxyethyl)phosphine hydrochloride (TCEP). Protein concentrations were measured using the BCA assay. After lysis, 50 μg of protein from each sample was precipitated with ice-cold acetone at −20 °C overnight. The precipitated proteins were then digested using the S-Trap digestion method (15). Disulfide bonds were reduced by adding 10 mM TCEP (Fisher), and protein alkylation was performed with 40 mM chloroacetamide (IAA) (Sigma). Samples were acidified by adding phosphoric acid to a final concentration of 1.2% and subsequently diluted six-fold with 90% methanol in 100 mM HEPES pH 8.5. The samples were added to an S-trap column (Protofi), and the column was washed twice with 90% methanol in 100 mM HEPES pH 8.5. Trypsin/Lys-C mixed enzyme (Pierce) was added to the S-trap column at a ratio of 1:25 (enzyme to protein ratio), and the digest reaction was allowed to continue for 2 h at 47 °C. Peptides were eluted in 40 μl of 50 mM HEPES pH 8.5 followed by 40 μl of 0.1% formic acid (FA) (Pierce) in water and 40 μl of 50/50 acetonitrile/water in 0.1% FA. Peptide concentrations were quantified using a tryptophan fluorescence assay. The peptides were then labeled with TMT 11-plex reagents (Thermo Fisher Scientific). The tandem mass tag (TMT)-labeled peptides were desalted using Oasis HLB cartridges and fractionated into 24 fractions by high-pH reversed-phase high-performance liquid chromatography (HPLC) with a 1260 BioLC system (Agilent) (Fig. 1*B*) (16).

### LC-MS/MS Analysis

Proteomic analysis was performed on the fractionated peptides using a high-resolution Orbitrap Exploris 480 mass spectrometer (Thermo Fisher Scientific) coupled with an Ultimate 3000 RSLC system (Dionex) as previously described with some modifications (17). Peptide samples were separated in a two-column system with a trap column (300 μm I.D × 5 mm length) and an analytical column (EASY-Spray C18, 75 μm I.D. × 50 cm length) with 120 min gradients from 8 to 30% acetonitrile at 300 nl/min. The column temperature was maintained at 60 °C using a column heater. The fractionated samples were resuspended in loading buffer (2% ACN and 0.1% FA) and loaded onto a column. A survey scan (m/z 350–1650) was performed at a resolution of 120,000 and an m/z of 200. The top-20 method was used to select precursor ions with an isolation window of 0.7 m/z. The MS/MS spectrum was acquired at an HCD-normalized collision energy of 32 and a resolution of 35,000 at m/z 200. The maximum ion injection times for the full and MS/MS scans were 20 ms and 100 ms, respectively.

### DIA-MS Proteomic Analysis

For data-independent acquisition (DIA)-MS-based proteomic analysis, islet samples were prepared from four individual donors. In two cases, islets from two donors were combined due to insufficient yield, resulting in three experimental sets. Protein extraction from islets was performed using a lysis buffer containing 4% SDS, 0.1 M Tris-HCl (pH 7.5), and 2 mM TCEP (Fisher). After sample lysis, protein concentration was quantified by the BCA assay. Subsequently, 50 μg of protein from each sample was precipitated overnight at −20 °C with ice-cold acetone. Protein digestion was performed according to the S-Trap digestion protocol described above (18). Peptide concentrations were measured by a tryptophan fluorescence assay. The peptides were acidified with 10% trifluoroacetic acid (TFA) and loaded onto custom-made styrene divinylbenzene reversed-phase sulfonate-StageTips according to previously described procedures (19). The StageTip was washed three times with 100 μl 0.2% TFA. The peptides were eluted with 0.2% TFA in 60% acetonitrile and 5% ammonium hydroxide in 80% acetonitrile. The desalted peptides were completely dried in a vacuum dryer and stored at −80 °C until further analysis.

Peptide samples were resuspended in 0.1% formic acid in HPLC-grade water containing indexed retention time (iRT) calibration peptides for DIA-MS analysis. Samples for DIA-MS were analyzed on a high-resolution Orbitrap Exploris 480 mass spectrometer (Thermo Fisher Scientific) coupled with an Ultimate 3000 RSLC system (Dionex). Peptides were separated in a two-column system with a trap column (300 μm I.D. × 5 mm, C18 3 μm, 100 Å) and an analytical column (IonOpticks Aurora C18, 75 μm I.D. × 60 cm) using a 140 min gradient from 5% to 50% acetonitrile at a flow rate of 300 nl/min. The column temperature was maintained at 60 °C. Full MS scans were acquired at a resolution of 120,000 at m/z 200 with a normalized AGC target of 300% and a maximum injection time of 25 ms over a mass range of m/z 350 to 1600. MS/MS spectra were acquired with a normalized AGC target of 2000%. A total of 40 variables isolation windows with a 0.5 m/z overlap covering the precursor m/z range of 350 to 1450 were applied (Table S2). MS/MS spectra were acquired over a scan range of m/z 145 to 1450 at a resolution of 15,000 at m/z 200 with automatic maximum injection time, and normalized collision energy was set to 30%.

### Database Search and Statistical Analysis for Proteomic Data

For the TMT-based discovery cohort, protein identification and quantification were carried out using Proteome Discoverer software (version 3.0) based on SEQUEST-HT search engine (Thermo Fisher Scientific). The acquired data was compared and searched against the UniProtKB HUMAN database (81,794 entries, version 2023-04). The search parameters were as follows: complete trypsin digestion; precursor mass tolerance of 10 ppm and MS/MS tolerance of 20 ppm; two max missed cleavage per peptide; false discovery rate (FDR) for all peptide-spectrum matches (PSMs) and protein under 1%, and performed by Percolator software package (version 3.05) using delta Cn (0.05), strict FDR (0.01), relaxed FDR (0.05), and PEP (0.05) settings; fixed modifications were TMT tag (N-term and Lys, + 229.163 Da) and carbamidomethylation (Cys, + 57.021 Da); and variable modifications were oxidation (Met, + 15.995 Da) and deamidation (Asn and Gln, + 0.984 Da). Peptide groups were assigned to logical protein groups based on the parsimony principle. The most confident centroid within 20 ppm of the expected mass of the reporter ions was used. The identified proteins with PSMs that contained all intensity values from all TMT channels were used to obtain the peptide quantification value.

For the DIA-based replication cohort, DIA files were processed with the direct DIA experimental analysis workflow in Spectronaut v19 (Biognosys AG) against the UniprotKB human protein sequence database (81,794 entries, version 2023-04) using default setting. Specific trypsin digestion was set with a maximum of two missed cleavages. A fixed carbamidomethyl modification of cysteine, and up to five variable modifications for oxidation of methionine and acetylation of the protein N-terminus were allowed. For the MS1 and MS2 mass tolerance, we used the default value (40 ppm) for Orbitrap MS in Spectronaut. PSM, peptide and protein FDR were set to 0.01. The precursors and proteins with a q-value of 0.01 from the mProphet model were considered for the identification of precursor and protein (20). All abundances were calculated based on the area under the extracted ion chromatogram (XIC) of all assigned fragments that passed filtering. The quantification of DIA data was performed by summing MS2 peak areas for each peptide with Spectronaut. Default settings of quantification were applied, with global normalization unenabled.

Statistical analysis of the proteomics data in the TMT and DIA datasets was conducted using Perseus software (Max Planck Institute of Biochemistry, Martinsried, Germany) to identify differentially expressed proteins (DEPs) across the experimental groups. In the TMT dataset, multiple group comparison analysis was performed to identify the group-specific expressed proteins using ANOVA with a nominal *p*-value <0.05. Two-sided t-tests were performed for pairwise comparisons of proteomes to detect DEPs with significant filtering criteria (*p* value < 0.05 and fold-change >1.2). DEPs were visualized using heatmaps and volcano plots to assess expression patterns (Fig. 1*C*).

In the DIA dataset, protein intensity values were log2 transformed, and protein groups that did not meet quantitation in 50% of at least one of the three conditions (Control, GLT, and GLT-PERKi) were filtered out to achieve the highest confidence due to the number of replicates used in this comparison (n = 3). Missing value imputation was performed based on the k-nearest neighbors (KNN) module with neighbor number of 15. After normalization by z-score transformation to remove donor effects from the data, multiple group comparison analysis was performed to identify the group-specific expressed proteins using ANOVA with a nominal *p*-value <0.05. For pair-wise analysis, a Welch’s *t* test was applied with a nominal *p*-value cutoff of <0.05 and |difference of z-score| > 1 (21).

### Bioinformatics and Functional Analysis

The DEPs were categorized into specific clusters based on their expression patterns. Various *in silico* analyses were then conducted on the clusters to elucidate their functional significance and pathway associations, as detailed below (Fig. 1*D*).

i ***Gene Ontology Analysis.*** GO analysis was used to investigate the functional roles of DEPs using the Database for Annotation, Visualization, and Integrated Discovery (DAVID) tool, focusing on three main domains: biological processes (BP), cellular components (CC), and molecular functions (MF). REVIGO (Reduce and Visualize Gene Ontology) was used to summarize and cluster GO terms based on semantic similarity.
ii ***KEGG Pathway Enrichment Analysis.*** KEGG pathway enrichment analysis was applied to identify the biological pathways associated with DEPs using the DAVID tool.
iii ***Protein-Protein Interaction Network Analysis.*** PPI network analysis was adopted to explore the interactions and functional relationships among DEPs using the STRING database and Cytoscape software (version 3.10.2).

### Western Blots

Total protein levels from homogenized islets were detected as previously described (3). We used the following primary antibodies: anti-PERK (Cell signaling #3192, 1:1000), anti-phospho-PERK (Cell signaling #3179, 1:1000), anti-PDX1 (Cell signaling #5679s, 1:1000), anti-KPNA4 (Santa Cruz #sc-390535, 1:500), and anti-γ-tubulin (Sigma #T6557, 1:5000).

### Immunocytochemical Staining

Fresh islets were dispersed into single cells and seeded onto sterile glass coverslips in Lab-TekTM Chamber Slides (Nunc, 30,108) and allowed to adhere overnight. Cells were fixed with 4% Paraformaldehyde (Biosolution, BP031a) in PBS, pH 7.4, for 10 min at room temperature. Permeabilization was achieved using 0.2% Triton X-100 in PBS for 10 min at room temperature. Non-specific binding was blocked with 5% BSA in PBS for 1 h at room temperature. Primary antibodies against FOXO1 (Cell Signaling, #2880), insulin (Invitrogen, #PA1-26938), PDX1 (Abcam, #ab47267), and TIA1 (Santa Cruz, #SC-166247) were diluted in 1% BSA/PBS and incubated with the cells overnight at 4 °C. Following primary antibody incubation, cells were incubated with secondary antibodies diluted in 1% BSA for 1 h at room temperature in the dark. The secondary antibodies used were Goat anti-Rabbit IgG Alexa Fluor 546 (Thermo Fisher, A-11035) and Goat anti-Mouse IgG Alexa Fluor 647 (Abcam, ab150115). Nuclei were counterstained using 2.5 μg/ml Hoechst 33,342 (Invitrogen, H3570) for 10 min. Coverslips were mounted on glass slides using SlowFade Diamond Antifade Mountant (Invitrogen, S36972). Imaging was then performed using a confocal laser scanning microscope (Leica TCS SP8, Inverted, Leica Microsystems). Fluorescence intensities and colocalization of PDX1 and TIA1 were analyzed using LAS X and ImageJ software (Fig. 1*E*).

### Quantitative Reverse Transcription PCR (qRT-PCR)

Total RNA was extracted from islets, cDNA was synthesized, and quantitative PCR was performed using the QuantStudio 5 Real-Time PCR System (Applied Biosystems) as published previously (3). Gene expression levels were normalized to *18S* rRNA. The primer sequences are presented in the Table S3.

### Statistical Analysis for *In Vitro* Data

All values are expressed as the mean ± standard error of the mean and median (interquartile range). For analyses involving three groups, one-way ANOVA with Bonferroni posttest and Tukey's multiple comparisons test was applied, and for two groups, the Student’s *t* test was used. *p* values less than 0.05 were considered statistically significant. Statistical analyses were performed using Perseus software (Max Planck Institute of Biochemistry), Prism nine software (GraphPad), and R (R Foundation for Statistical Computing).

## Results

### Characteristics of Participants and Isolated Islets

Pancreatic tissues were collected from 22 participants (mean ± SD age, 55 ± 14 years; six males [27%]) undergoing surgery for localized pancreatic lesions, including 11 cystic neoplasms, seven neuroendocrine tumors (including one case of insulinoma), and four pancreatic ductal adenocarcinomas. None of the participants had received anticancer therapy, systemic glucocorticoids, or vasopressors within 3 months prior to surgery (Table S4).

The cold ischemic time was 2.8 ± 4.7 h. The median [range] tissue weight subjected to islet isolation was 0.70 [0.18–5.01] g, and the islet yield per individual was 240 [45–850] IEQs. In addition, islets from two deceased, non-diabetic donors distributed by the Integrated Islet Distribution Program (IIDP) were included for validation studies (Table S4).

Most lesions in the SNUH participants primarily involved the exocrine pancreas. Although the endocrine and exocrine compartments are functionally distinct and only non-diabetic participants were enrolled, exocrine pathology and associated changes, such as local inflammation, could theoretically influence islet physiology. Notably, insulinomas that autonomously secrete excess insulin may affect adjacent non-pathologic tissue from which islets are isolated. To minimize these potential *in vivo* influences prior to isolation, all isolated islets were incubated overnight before initiation of experimental treatments. In addition, all available pathological reports were carefully reviewed before downstream analyses, confirming that there was no histological evidence of overt inflammatory changes in the surrounding pancreatic tissue of primary lesions.

### Proteome Characterization: TMT-Based Analysis

The number of islets available from a single donor was often limited, resulting in an unequal distribution of samples across treatment conditions, with an emphasis on GLT ± PERKi paired comparisons (Table S4). Further, to ensure sufficient protein yield for mass spectrometry, islets from donors with low yield were pooled prior to protein extraction. Consequently, a total of nine samples from seven individuals were prepared for TMT quantitative proteomic analysis, including two control samples, 3 GLT samples, and 4 GLT + PERKi treatment (Fig. 1, *A* and *B*). Although the control condition included only two protein samples, these samples collectively represented islets from four independent donors, indicating four biological replicates.

High-resolution LC-MS/MS analysis resulted in the identification of 5945 proteins at a 1% FDR at protein levels (Table S5). The identified proteins showed an average sequence coverage of 15.3%, with a median sequence coverage of 10%. The number of unique peptides per protein ranged from 0 to 197, with a mean of six. Among the total identified proteins, 5513 were quantified without missing values and used for further data analysis (Table S6).

To assess the quality of proteomic data, we generated profile plots showing the distributions of log-transformed intensities across TMT channels, which indicated consistent protein expression levels across groups without substantial shifts in median values (Fig. S1*A*). In addition, the average coefficient of variation for protein abundance within each experimental group was less than 10%, indicating good reproducibility (Fig. S1*B*).

The quantified protein abundances spanned four orders of magnitude, with just 292 proteins contributing to half of the total protein mass (Fig. S1*C* and Table S6). Since these 292 proteins predominantly drive the variance within the dataset of 5513 quantified proteins, they have a substantial impact on the overall distribution. In contrast, the remaining ∼4000 proteins account for just 25% of the total protein mass, reflecting a markedly skewed distribution in protein abundance (Fig. S1*D*). This imbalance suggests that the overall variability in the proteomics dataset is largely driven by this small subset of highly abundant proteins, as evidenced by the principal component analysis (PCA), which shows limited separation between groups (Fig. S2).

Notably, these highly abundant proteins showed significant enrichment for pancreas-specific expression based on the Human Protein Atlas database (*p* = 4.4 × 10^−29^) (Table S7). In addition, the abundant expression of the β-cell–specific marker insulin indicated the high quality of the samples (22), and the treatment-dependent expression patterns were consistent with our previous finding that PERKi reverses GLT-induced suppression of insulin levels (inset of Fig. S1*D*) (3).

### Quantitative Analysis of DEPs by GLT-Induced Stress and PERK Attenuation

**Fig. 2:**
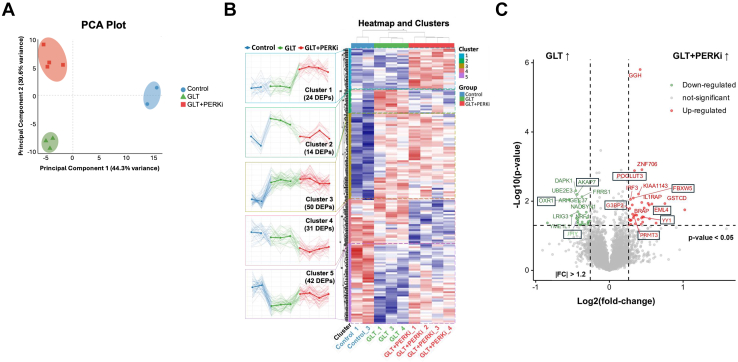
**Differential Expression Proteins across Control, GLT, and GLT + PERKi Groups of the TMT-Based Datasets.***A*, PCA of 161 DEPs identified among the three treatment groups by ANOVA (*p* < 0.05). The PCA plot shows distinct clustering of the control (*blue*), GLT (*green*), and GLT + PERKi (*red*) groups, with PC1 and PC2 explaining 44.3% and 30.6% of the variance, respectively. *B*, heatmap showing the expression profiles of the 161 DEPs. Hierarchical clustering divided the DEPs into five distinct clusters, with representative *line* plots for each cluster depicting average expression trends. Cluster 2 (14 proteins) and Cluster 5 (42 proteins) represent proteins whose GLT-induced upregulation and downregulation respectively, were reversed by PERKi. *C*, Volcano plot comparing GLT + PERKi *versus* GLT. The y-axis represents the −log10(*p*-value), and the x-axis represents the log_2(fold change). Colored dots indicate those that meet the dual criteria of a *p*-value <0.05 and a fold change threshold of ±1.2. Proteins downregulated (*green*) and upregulated (*red*) in the GLT + PERKi group are depicted. Proteins in Cluster 2 and Cluster 5 are highlighted as *black box*, labeling representative DEPs. ANOVA, analysis of variance; DEP, differentially expressed protein; GLT, glucolipotoxicity; PC, principal component; PCA, principal component analysis; PERKi, pancreatic endoplasmic reticulum kinase inhibitor.

To identify proteins exhibiting significant differences in abundance among the three experimental conditions, an ANOVA was conducted on the quantified protein dataset. A total of 161 proteins showed significant differences, as listed in Table S8. This subset represents approximately 3% of the total 5513 quantified proteins, highlighting that only a small fraction of the proteome exhibited statistically significant changes under the experimental conditions. As expected, the PCA plot based on the 161 significantly regulated proteins revealed clear separation among the three groups. Principal Component 1, which accounted for 44.3% of the total variance, separated the control group from the other groups. Principal Component 2, which accounted for 30.6% of the variance, further distinguished the GLT + PERKi group from the GLT group (Fig. 2*A*).

The heatmap demonstrated distinct expression patterns corresponding to each experimental condition, which were categorized into five clusters (Fig. 2*B*). Cluster 2 and Cluster 5 were of particular interest due to their association with both GLT and PERKi treatment. In Cluster 2, 14 proteins were significantly upregulated in the GLT group compared to the control, with their expression levels reduced by PERKi, returning close to control levels. Conversely, 42 proteins in Cluster 5 were markedly downregulated by GLT, with their expression partially restored by PERKi.

Figure 2*C* highlights the effect of PERK attenuation under GLT-induced stress, irrespective of the changes caused by GLT. Among a total of 59 DEPs identified using a fold change threshold of ±1.2 in a pairwise comparison between the GLT and GLT + PERKi groups, 25 proteins were significantly downregulated, and 34 were upregulated by PERKi (Table S9). These DEPs were partially overlapping with Clusters 2 and 5, respectively.

### Functional Clustering Reveals Intracellular Transport and Enzymatic Catabolism as Main β-Cell Responses to PERK Attenuation Under GLT Stress

**Fig. 3:**
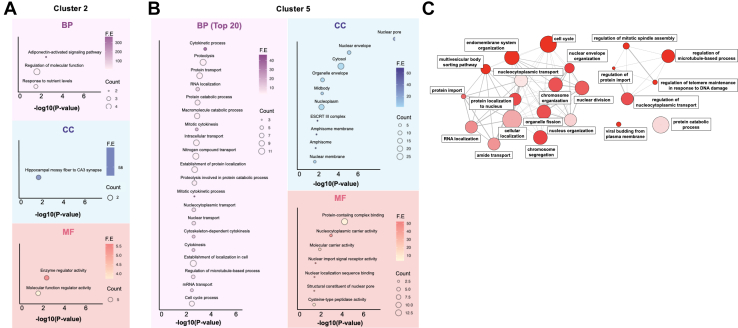
**GO Enrichment Analysis of DEPs in Clusters 2 and 5 of the TMT-Based Datasets.** Gene ontology (GO) enrichment analysis was conducted based on the three GO domains: Biological Process (BP), Cellular Component (CC), and Molecular Function (MF). Fold enrichment (F.E.) is represented by color intensity, and the number of proteins contributing to each term (“Count”) is represented by the size of the *circles*. *A*, GO analysis of Cluster 2 (*B*) GO analysis of Cluster 5. For BP terms, only the top 20 terms are shown. *C*, network visualization of enriched BP terms in Cluster 5 using REVIGO. Functionally similar GO terms are grouped based on semantic similarity, with node size corresponding to fold enrichment.

GO enrichment analysis of Clusters 2 and 5 revealed distinct BP, CC, and MF terms, highlighting responses to GLT and PERKi in human islets. Cluster 2 was enriched in pathways associated with molecular regulation in response to nutrient levels, particularly those involving enzyme activity (Fig. 3*A*).

In the meanwhile, Cluster 5 was enriched in key GO terms related to “cytokinetic process,” “proteolysis,” and nucleocytoplasmic transport (NCT) including BP terms such as “protein transport,” “RNA localization,” CC terms like “nuclear pore,” “nuclear envelope,” and MF terms including “nucleocytoplasmic carrier activity” (Fig. 3*B*). Furthermore, REVIGO analysis revealed a tightly interconnected network of GO terms, with “protein localization to the nucleus” and “NCT” emerging as central nodes (Fig. 3*C*).

The collective results from Clusters 2 and 5 also suggest autophagic activity as indicated by BP terms such as “response to nutrient levels”, “proteolysis”, MF terms like “enzyme regulator activity,” “protein-containing complex binding,” and CC terms including “cytosol,” “organelle envelope” (23, 24). These findings align with our previous finding that autophagy mediates the protective effects of PERKi against GLT-induced stress in β-cells (3).

### Pathway and Network Analyses Identify NCT as a Central Mechanism of PERKi

**Fig. 4:**
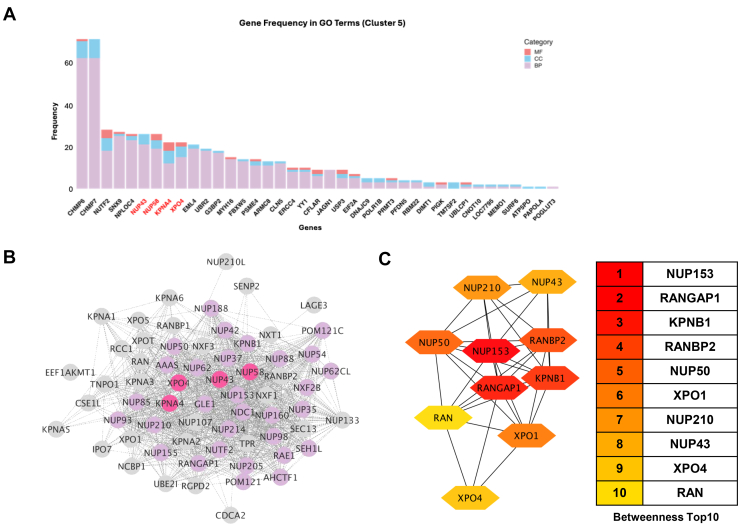
**Functional and Interaction Analysis of Cluster 5 Proteins of the TMT-Based Datasets.***A*, Gene frequency plot demonstrates the contribution of individual genes to the biological processes categorized into Gene ontology (GO) terms within Cluster 5. Categories are color-coded as follows: Molecular Function (MF, *red*), Cellular Component (CC, *blue*), and Biological Process (BP, *purple*). *B*, A protein–protein interaction (PPI) network constructed using nucleocytoplasmic transport (NCT)-related proteins (NUP43, NUP58, KPNA4, and XPO4) identified through KEGG pathway analysis (Table 1), illustrating the relationships among key proteins. *Nodes* represent proteins, and *edges* indicate interactions. *C*, network rank highlighting the top 10 proteins based on betweenness centrality from the PPI network shown in (*B*).

Figure 4*A* displays a gene frequency map illustrating the number of occurrences of each gene across enriched GO terms in Cluster 5. This highlights the relative contribution of individual proteins to functional enrichment. KEGG pathway analysis revealed NCT as the only significantly enriched pathway in Cluster 5 (Table 1).

**Table 1:** KEGG pathway enrichment analysis for Cluster 5 of the TMT-based dataset

KEGG ID	KEGG pathway	Count	%	*p*-value	Genes	List total	Pop Hits	Pop total	Fold Enrich-ment
hsa 03,013	Nucleocytoplasmic transport	4	9.76	0.002	*XPO4, KPNA4, NUP43, NUP58*	22	108	8662	14.58
hsa 04,217	Necroptosis	3	7.32	0.057	*CFLAR, CHMP6, CHMP7*	22	160	8662	7.38

“Count” indicates the number of DEPs associated with each KEGG pathway. The “%” column represents the percentage of DEPs related to the pathway relative to the total proteins annotated with that pathway in the database. “Genes” lists the gene symbols corresponding to the DEPs. “List Total” shows the total number of proteins used in the analysis for each pathway. “Pop Hits” indicates the number of genes associated with the KEGG pathway in the entire database, and “Pop Total” represents the total number of genes in the database. “Fold Enrichment” is calculated by comparing the observed frequency of a KEGG pathway in the DEPs to its expected frequency in the database, providing a measure of over-representation.

The nucleus is enclosed by a double-membrane nuclear envelope that separates the nucleoplasm from the cytoplasm. This barrier is perforated by portals known as nuclear pore complexes (NPCs), which mediate NCT. The NPC is a highly selective, bidirectional transporter that regulates the trafficking of a wide range of protein and ribonucleoprotein cargoes between the nucleus and cytoplasm (25). Consistently, the NCT pathway identified in our analysis was enriched for key regulators of NCT, including the importin family member KPNA4 and the exportin XPO4, as well as structural components of the NPC such as NUP43 and NUP58. In contrast, KEGG pathway analysis of Cluster 2 revealed no significantly enriched pathways.

To further investigate the functional significance of these NCT-related proteins, a PPI network was constructed using them (Fig. 4*B*). In the network ranking based on betweenness centrality within the PPI network, XPO4 and NUP43 were ranked among the top 10, indicating their role as crucial hubs with high connectivity and centrality (Fig. 4*C*). The overall bioinformatic workflow with DEPs, particularly for Cluster 5, is summarized in Figure S3.

### Validation of Key Findings in an Independent Replication Cohort Using DIA-MS

**Fig. 5:**
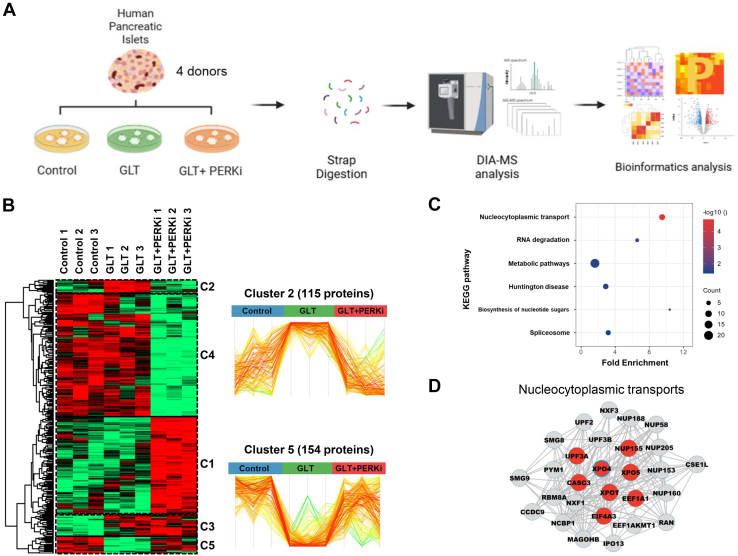
**Validation of the Islet Proteome Using DIA-MS in an Independent Replication Cohort.***A*, Experimental workflow for the replication study. Human pancreatic islets from four additional donors were analyzed *via* DIA-MS to validate the findings from the discovery phase. *B*, heatmap and expression profiles. Hierarchical clustering of significant proteins (ANOVA *p*-value <0.05) reveals consistent proteomic changes. Cluster 2 (115 proteins) and cluster 5 (154 proteins) represent groups of proteins whose expression was significantly modulated by GLT and restored by PERKi. *C*, KEGG pathway enrichment analysis. Dot plot highlighting significantly enriched pathways. Nucleocytoplasmic transport emerged as a top-ranking term in both GOBP and KEGG analyses for Cluster 5. *D*, Protein–protein interaction (PPI) network of the NCT pathway. The network illustrates the functional connectivity of the pathway. Proteins colored red indicate those identified in our experimentally derived Cluster 5 (including CASC3, EIF4A3, XPO4, XPO5, XPOT, NUP155, PCK1, UPF3A, and EEF1A1), which form the core of the interaction network. Proteins in *gray* represent other known members of the NCT pathway included for context. The consistent expression pattern of XPO4 across both discovery and replication experiments highlights its robustness as a marker.

To address concerns regarding sample size and the pooling strategy, we analyzed an independent replication cohort of human islets from four additional donors using DIA-MS (Fig. 5*A* and Table S4). ANOVA identified a total of 2436 proteins showing significant differences across the three experimental conditions (Table S10). Hierarchical clustering revealed distinct expression patterns corresponding to each condition, which were categorized into five clusters (Fig. 5*B*), consistent with the results of the TMT-based discovery analysis (Fig. 2*B*).

Next, 154 proteins of Cluster 5, characterized by downregulation under GLT and recovery with PERKi, were subjected to further analyses. Enrichment analysis based on GOBP and KEGG pathway demonstrated a significant enrichment of the “nucleocytoplasmic transport” term (Fig. 5*C*). This enrichment was driven by a core set of proteins, including CASC3, EIF4A3, XPO4, XPO5, XPOT, NUP155, PCK1, UPF3A, and EEF1A1. To further examine the functional relationships among these candidates, we constructed a PPI network for the NCT pathway. Notably, these proteins formed a highly interconnected core within the broader network of transport-related proteins (Fig. 5*D*). Particularly, XPO4 exhibited an expression pattern identical to that observed in the TMT-based discovery cohort, demonstrating successful replication across independent datasets. Collectively, these findings confirm that modulation of NCT represents a consistent biological response to GLT and PERK inhibition in human islets.

### NCT Modulation of Transcription Factors by PERKi

To functionally validate the NCT pathway involved in the PERKi action, we performed a series of experiments focusing on two representative transcription factors in β-cells: PDX1 (pancreatic and duodenal homeobox 1) and FOXO1 (forkhead box O1). Both transcription factors regulate a wide range of critical cellular functions in pancreatic β-cells in response to metabolic stress (26, 27).

**Fig. 6:**
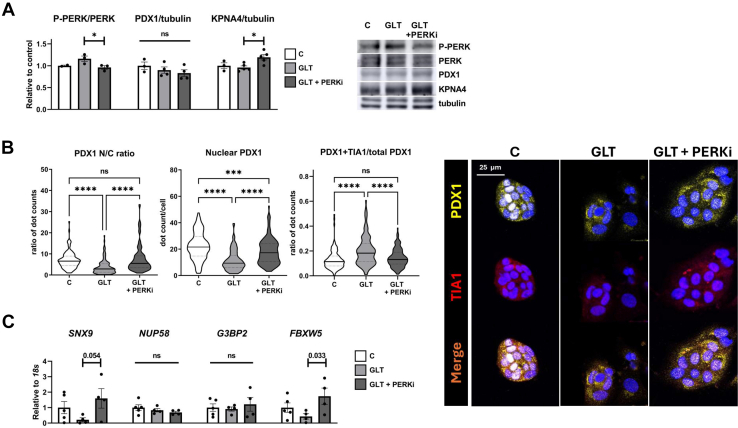
**Modulation of PDX1 NCT by PERKi in Human Islets under GLT.***A*, Western blot analysis of P-PERK, PERK, PDX1, and KPNA4 levels, along with representative blots. *B*, immunocytochemical analysis of PDX1 staining and its co-localization with the stress granule marker TIA1. Two independent experiments were conducted, and an average of 554 ± 123 cells were analyzed per treatment group. Representative confocal images show PDX1 (*yellow*), TIA1 (*red*), and nuclei (*blue*). *C*, quantitative RT-PCR analysis of selected PDX1 target genes from Cluster 5 of the TMT-Based Datasets. *p*-values between indicated groups are labeled. Data are expressed as the mean ± standard error of the mean (SEM) in (*A*) and (*C*), and as the median ± interquartile range (IQR) in (*B*). Each *dot* in (*A*) and (*C*) indicates a biological replicate. ∗, *p* < 0.05; ∗∗∗, *p* < 0.001; ∗∗∗∗, *p* < 0.0001 by one-way ANOVA with Tukey's multiple comparisons test. *C*, control; FBXW5, F-Box and WD Repeat Domain Containing 5; GLT, glucolipotoxicity; G3BP2, G3BP stress granule assembly factor two; KPNA4, karyopherin subunit alpha 4; N/C ratio, ratio of nucleus-to-cytoplasm; NCT, nucleocytoplasmic transport; NUP58, nucleoporin 58; ns, no significant difference; PDX1, pancreatic and duodenal homeobox 1; PERKi, PERK inhibitor; SNX9, sorting nexin 9; TIA1, T-cell intracellular antigen-1; SNX9, sorting nexin nine.

First, chronic exposure to palmitate has been shown to cause cytoplasmic sequestration of PDX1 with colocalization to the stress granule marker TIA1 (T-cell restricted intracellular antigen 1), thereby impairing its nuclear translocation and transcriptional activity (28). Based on this observation, we investigated the NCT of PDX1 in human islets under GLT conditions as a functional readout of PERKi-mediated modulation of NCT. PERK attenuation was confirmed by reduced PERK phosphorylation through Western blotting. PDX1 protein levels were not significantly altered by PERK inhibition, whereas KPNA4 levels significantly increased (*p* < 0.05) (Fig. 6*A*). KPNA4 is an importin-α family member that facilitates cargo transport via the NPC (29).

Having established that PERKi does not alter total PDX1 protein abundance but modulates components of the nuclear import machinery, we next examined PDX1 subcellular localization by immunocytochemistry (Fig. 6*B*). GLT significantly reduced the nuclear-to-cytoplasmic ratio of PDX1 staining, whereas PERKi treatment restored this ratio (both *p* < 0.001). A similar pattern was observed for nuclear PDX1 intensity. Consistently, GLT induced a marked increase in cytoplasmic PDX1 colocalized with TIA1, an effect that was reversed by PERKi treatment (both *p* < 0.001). These findings were reproduced in mouse islets (Fig. S4).

Next, we assessed whether PERKi-induced modulation of PDX1 NCT was accompanied by changes in PDX1-dependent transcriptional activity. Using TFlink (https://tflink.net/), we identified transcriptional targets of PDX1 within Cluster 5 of the TMT dataset, of which the four most frequently represented genes were selected for validation by qRT-PCR (Table S11). As a result, PERKi treatment appeared to increase *FBXW5* mRNA expression relative to *18S rRNA* in human islets under GLT conditions (*p* = 0.033; Fig. 6*C*).

Collectively, these validation experiments suggest that PERKi restores the nuclear localization and functional activity of PDX1 under GLT conditions, potentially through modulation of the NCT machinery. In contrast, neither high-dose PERKi, which lacks β-cell–enhancing effects (9, 12), nor a structurally distinct PERK inhibitor, HC-5404 (14) at an equivalent dose to low-dose PERKi, altered PDX1 NCT (Figs. S5 and S6).

**Fig. 7:**
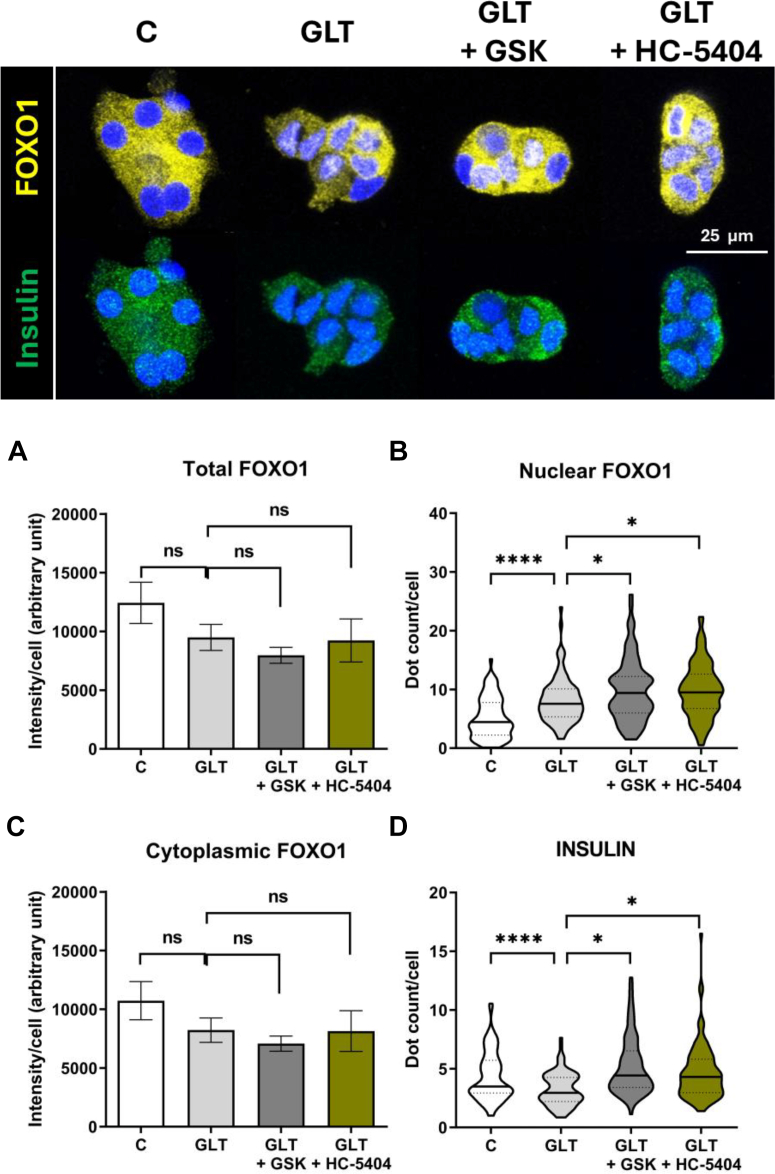
**Modulation of FOXO1 NCT by PERKi in Mouse Islets under GLT.** Immunocytochemical analysis of FOXO1 was performed in dispersed mouse islet cells. FOXO1 staining was quantified in insulin-positive cells across two independent experiments, with an average of 959 ± 78 cells analyzed per treatment group. Structurally distinct two PERK inhibitors were tested, GSK2606414 (40 nM) and HC-5404 (160 nM). Representative confocal images show FOXO1 (*yellow*), insulin (*green*), and nuclei (*blue*). Quantitative analyses are shown for (*A*) total FOXO1, (*B*) nuclear FOXO1, (*C*) cytoplasmic FOXO1, and (*D*) insulin staining. Data are expressed as the mean ± standard error of the mean (SEM) in (*A*) and (*C*), and as the median ± interquartile range in (*B* and *D*). ∗, *p* < 0.05; ∗∗∗∗, *p* < 0.0001 by one-way ANOVA with Tukey's multiple comparisons test. C, control; FOXO1, forkhead box O1; GLT, glucolipotoxicity; GSK, GSK2606414; NCT, nucleocytoplasmic transport; ns, no significant difference.

The second transcription factor examined was FOXO1. FOXO1 dephosphorylates under metabolic stress, resulting in its nuclear accumulation and activation of target gene transcription (30). Notably, XPO4, consistently identified as an enriched protein across our independent proteomic datasets, is predicted to be a transcriptional target of FOXO1 (https://tflink.net/). Neither GLT nor PERK inhibition significantly altered the total FOXO1 staining intensity in insulin-positive mouse cells (Fig. 7*A*). In contrast, GLT induced an approximately 70% increase in nuclear FOXO1 intensity (Fig. 7*B*). Unlike PDX1, this GLT-induced nuclear accumulation was not reversed but rather modestly enhanced (∼25%) by both PERK inhibitors, GSK2606414 and HC-5404, at low doses (all *p* < 0.05). Cytoplasmic FOXO1, which represents the predominant subcellular pool, was not significantly affected by PERK inhibition (Fig. 7*C*). As expected, insulin immunofluorescence intensity was significantly reduced by GLT, which was restored by either PERK inhibitors (all *p* < 0.05, Fig. 7*D*). Taken together, low-dose PERK inhibition modulates the NCT of transcription factors in β-cells; however, these effects are heterogeneous.

## Discussion

This study offers comprehensive proteomic insights into the mechanisms by which PERK attenuation mitigates GLT-induced β-cell dysfunction. To address the inherent biological challenges associated with human primary islet research, we employed a multi-faceted proteomics strategy. Human islets exhibit substantial inter-individual donor heterogeneity and intra-islet cell heterogeneity (31, 32), both of which can introduce significant variability and obscure true biological signals. To ensure the reliability of our findings, we complemented our initial discovery phase conducted via TMT-based quantification with an independent replication cohort analyzed through the DIA method. This orthogonal MS platform approach was specifically designed to identify consistent protein-level changes that transcend individual donor variability. By focusing on proteomic signatures consistently observed in both datasets, we demonstrated that PERK inhibition exerts a robust and reproducible effect on the islet proteome under GLT conditions. The successful validation of the key pathway, the NCT machinery, across independent cohorts and acquisition methods strongly supports the biological relevance of this pathway and argues against cohort-specific artifacts.

Building on this cross-platform validation, subsequent analyses were designed to functionally interrogate the NCT pathway itself, rather than to confirm individual DEPs. Within this framework, the transcription factors PDX1 and FOXO1 were selected as well-characterized and biologically relevant exemplars, and our data highlight modulation of NCT as a plausible mechanism underlying the protective effects of PERKi in human islets exposed to diabetes-associated metabolic stress. Importantly, these effects appear to be heterogeneous, depending on the PERK inhibitor scaffold and the specific NCT targets involved, raising the possibility that PDX1 NCT modulation may constitute an off-target effect of GSK2606414.

NPCs are portals mediating NCT. Each NPC consists of multiple copies of ∼30 different proteins, collectively termed nucleoporins (29). Among the proteins enriched in the KEGG pathway analyses across the two datasets, NUP43, NUP58, and NUP155 were identified as core nucleoporins. Notably, NUP155 has been implicated in atrial fibrillation, underscoring the physiological importance of intact NPC function (33).

In addition to the NPC, NCT is regulated by soluble transporters that either deliver cargo to the NPC or modulate its translocation across the NPC (25). Among the proteins enriched in the KEGG pathway analyses, XPO4, XPO5, XPOT, and KPNA4 fall into this category. As a member of the importin-α family, KPNA4 recognizes classical nuclear localization signal (NLS) (29), which is present in both PDX1 and FOXO1 (34, 35). Thus, the increased KPNA4 levels following PERKi treatment may have facilitated the nuclear import of these transcription factors. In contrast, XPO4 is a bidirectional nuclear transport receptor whose cargo recognition mechanisms are not fully established yet (29). Given that XPO4 is predicted to be a transcriptional target of FOXO1, it may participate in PERKi-mediated metabolic effects, a possibility that warrants further investigation.

We observed that PERKi treatment enhanced the nuclear transport of both PDX1 and FOXO1. The significance of NCT in β-cell biology is well established (36, 37). PDX1 represents a key NCT target in the context of GLT, with both high glucose and saturated fatty acids affecting its localization and transcriptional activity (28, 38). Therefore, restoring PDX1 function, including promotion of β-cell growth and maintenance of identity, has been a therapeutic target in diabetes (27).

In contrast, FOXO1 primarily orchestrates stress-adaptive, energy-conserving responses (26). Its nuclear accumulation in response to cellular stress is often regarded as a protective adaptation (39), whereas reduced nuclear PDX1 is typically interpreted as a maladaptive response. Notably, FOXO1 and PDX1 have been reported as reciprocal transcriptional targets, implying a potential cross-regulatory mechanism that coordinates their activity under metabolic stress (26). Consistent with this notion, Cluster 5 includes genes predicted to be regulated not only by PDX1 or FOXO1 (Table S11), but also by multiple transcription factors involved in β-cell identity, metabolism, and stress responses, including MAFA, HNF1A, and NRF1 (TFlink). Although our functional validation focused on only two transcription factors, PERK inhibition–mediated modulation of NCT is unlikely to be restricted to PDX1 and FOXO1 but may broadly influence transcriptional networks.

Previous omics analyses using human islets have advanced our knowledge on the molecular mechanisms underlying diabetes-related β-cell dysfunction (40, 41). For example, proteomic analyses of islets exposed to high glucose have revealed enrichment of GO terms related to glucose metabolism, apoptosis, and macromolecular complex assembly. Notably, one of the identified proteins was NXF1 (nuclear RNA export factor 1), a transporter that regulates the NCT of RNA (42). In contrast, pathway analyses of transcriptomic and proteomic changes induced by palmitate have highlighted alterations in lipid metabolism, oxidative stress, amino acid metabolism, and cell cycle pathways (43). Another study identified autophagy as a key mediator of adaptive endoplasmic reticulum stress signaling in response to palmitate (44).

Since treatment of type 2 diabetes typically begins under conditions of chronic hyperglycemia associated with obesity, we used both high glucose and palmitate (*i.e.,* GLT) to mimic the cellular environment in which PERKi is expected to act. Across our two independent proteomic analyses, we consistently identified two DEPs under GLT conditions, suggesting potential relevance in this context that warrants further investigation: fatty acyl-CoA reductase 1 (upregulated by GLT) and dimethyladenosine transferase (downregulated by GLT). Notably, in the only published omics study to date examining GLT conditions in human islets—reporting 1855 differentially expressed transcripts (45)—transcriptional changes in these two genes were not significant. Indeed, we found limited overlap between the transcriptomic changes reported and the DEPs identified in our pairwise comparisons between control and GLT conditions (Tables S9 and S12, and Fig. S7). Moreover, approximately half of the overlapping DEPs exhibited opposite directionality compared to the transcriptomic changes, likely due to post-transcriptional regulation, compensatory cellular responses, and differences in experimental design. This mismatch underscores the importance of our proteomic analysis, as proteins represent the actual functional units within cells.

Meanwhile, multi-omics profiling of islet pools from 39 patients with type 2 diabetes also revealed autophagy and NCT as significantly enriched pathways compared to those from non-diabetic individuals (46). As suggested by our current GO enrichment analysis (Fig. 3) and previous work (3), autophagic activity can be modulated by PERK inhibition, supporting its potential as a therapeutic strategy for preserving β-cell function in type 2 diabetes. Notably, among the target genes of PDX1 and FOXO1, *FBXW5*—a gene significantly upregulated by PERKi (Fig. 6*C* and Table S11)—is involved in nutrient-sensitive autophagy (47), indicating that enhanced NCT may mediate PERKi-induced autophagic regulation.

In our study, human islets were isolated from non-diabetic donors to exclude the effects of diabetes-associated genes. Particularly, islets for the proteomic analysis were obtained from living donors of the same ethnicity, helping to minimize intrinsic variability and reduce confounding effects related to genetic background or donor status. Compared to islets from deceased organ donors, those from living donors more accurately reflect physiological conditions, as they are less affected by metabolic disturbances potentially associated with brain death and postmortem autolysis of pancreatic tissue (48). Furthermore, pancreas samples obtained at the same institute were exposed to only a short duration of ischemia, thereby better preserving islet integrity. By using these islets, the study aimed to minimize biological variability and potential confounding factors.

Despite these advantages of a strategy involving living donors, it has inherent limitations. First, donors have underlying pancreatic disease, which may influence islet samples. Although clinical pancreatitis was an exclusion criterion and islets were isolated only from normal-appearing tissue, microscopic lesions or tumor-associated inflammatory responses cannot be completely ruled out and may potentially affect β-cell physiology. In particular, in cases of insulinoma, autonomous insulin hypersecretion from the tumor may have induced atrophic changes in surrounding normal β cells. To mitigate these *in vivo* influences, we implemented an *ex vivo* stabilization incubation following islet isolation before initiating experimental treatments. Additionally, islets from each donor were allocated to treatment groups as evenly as possible to ensure within-donor comparisons, which would help identify individual outlier results and interpret them in the context of donor-specific disease states. However, this method was not always feasible due to limited islet yield, which is discussed below as the second limitation of living donors.

The yield of islets from a single living donor is inherently limited. Islets were isolated from the resection margin, excluding the pathological lesion, and the resection margins are typically very small to preserve sufficient normal pancreatic tissue for patient safety. This constraint precluded the generation of complete experimental sets for every donor, and in some cases, pooling of samples was required to obtain sufficient protein for proteomic analyses. These caused reduced statistical power, and sensitivity analyses—such as exclusion of data from the participant with insulinoma—were not practically feasible.

We acknowledge that the 2 to 4 experimental replicates in the TMT-based discovery phase, which involved pooled samples, may be insufficient to achieve robust statistical power. However, the key findings were independently reproduced in the DIA-MS analysis using balanced and independent sample allocation, consistently converging on NCT as a central pathway of PERK inhibition and identifying XPO4 as an enriched DEP. This reproducibility partially mitigates the statistical limitations imposed by restricted islet yield. Future studies using larger numbers of donors-ideally without pancreatic diseases-will be necessary to more precisely delineate gene-specific effects and to further clarify the links between PERK inhibition, NCT regulation, and β-cell function.

This study has additional limitations. Although our proteomic analyses provided valuable insights, they primarily identified associations rather than direct causal relationships. Further in-depth investigations are warranted to clarify the causal role of NCT, particularly involving XPO4, its candidate cargoes, and the mediating mechanisms. Finally, while PERKi reversed some molecular changes *in vitro*, its *in vivo* mechanisms have yet to be clarified. Further animal studies are needed for this issue.

In summary, through integrative proteomic and functional analyses, this study highlights the modulation of NCT as a therapeutic mechanism by which PERKi reverses GLT-induced β-cell dysfunction, acting on multiple NCT targets with heterogeneous effects that depend on both the transport cargoes and the chemical structures of the PERK inhibitors. Elucidating how diverse NCT pathways are regulated may provide a foundation for future research into the application of PERKi in diabetes management by restoring β-cell function.

## Data Availability

The clinical data is not publicly available due to data privacy regulations and institutional policies. However, de-identified data may be made available from the corresponding author, Professor Hye Seung Jung (Department of Internal Medicine, Seoul National University Hospital, Email: jungjhs@gmail.com), upon reasonable request and with appropriate institutional and ethical approvals. The mass spectrometry proteomics data have been deposited to the ProteomeXchange Consortium via the PRIDE (49) partner repository with the dataset identifier PXD066613 and PXD074587. Spectronaut (.sne) files containing annotated spectra and associated metadata were also deposited to support verification of single-peptide identifications.

## Supplemental Data

This article contains supplemental data (50, 51, 52).

## Conflict of Interest

The authors declare that they have no conflicts of interest with the contents of this article.
