# Supplementary material for: Proteomic Analysis on Human Islets Suggests Nucleocytoplasmic Transport as a Mechanism of PERK Attenuation Effects in Diabetes
Source: Mol Cell Proteomics. 2026 May 18;25(6):101588. doi: 10.1016/j.mcpro.2026.101588 (PMC13277438; doi:10.1016/j.mcpro.2026.101588)
Supplement: Figures S1–S7 [file mmc1.docx]

**Supplementary Figures**


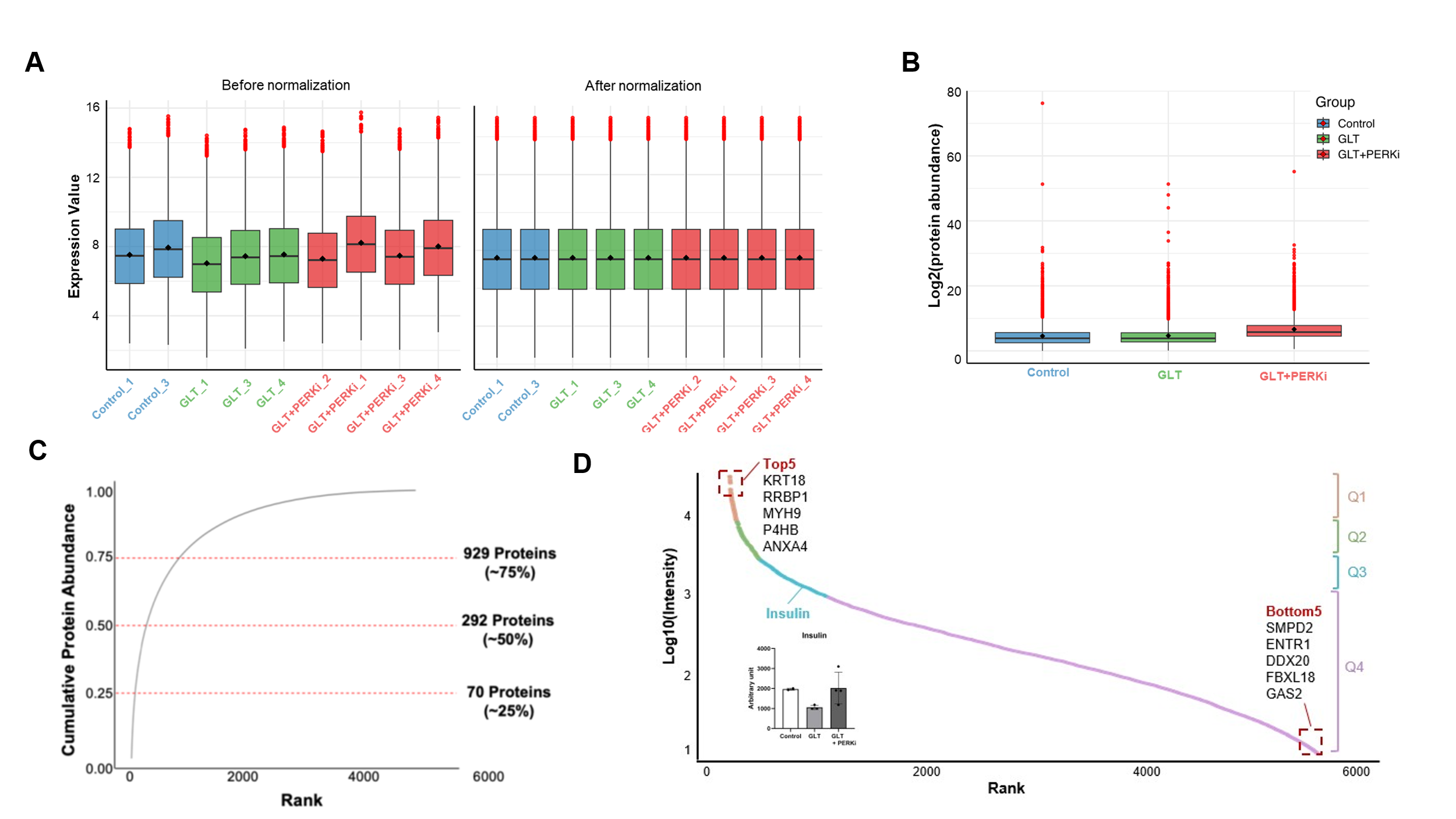


**Figure S1. Evaluation of TMT-based proteomic data quality and distribution.**

(A) Distribution of log-transformed protein intensities across TMT channels.

(B) Coefficient of variation (CV) within each group.

(C) Cumulative abundance plot

(D) Protein abundance distribution across the proteome. *INS* expression is shown in the inset.


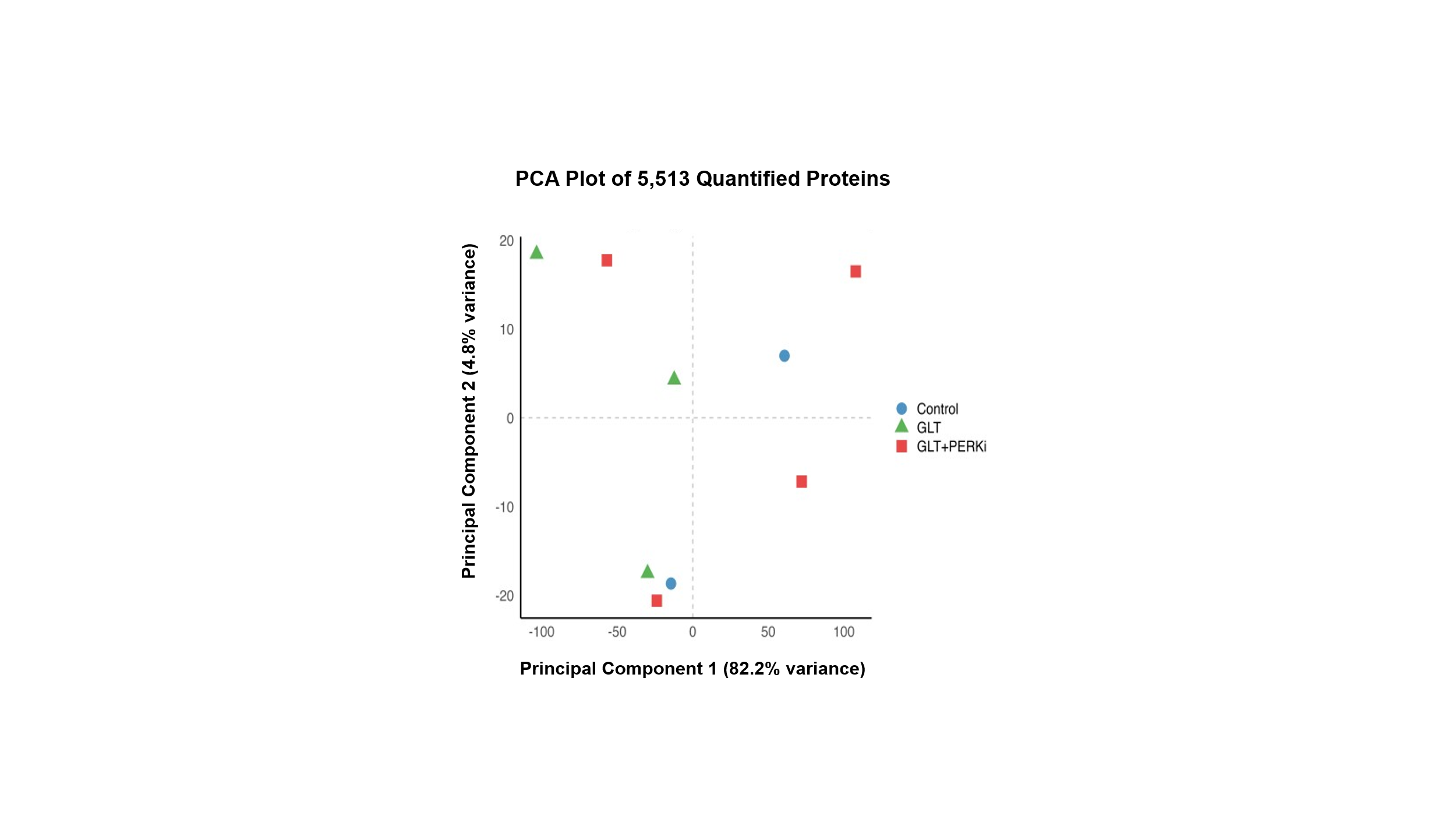


**Figure S2. Principal component analysis (PCA) of the TMT-based proteomic dataset.**

PCA based on the 5,513 quantified proteins shows limited separation between experimental groups.


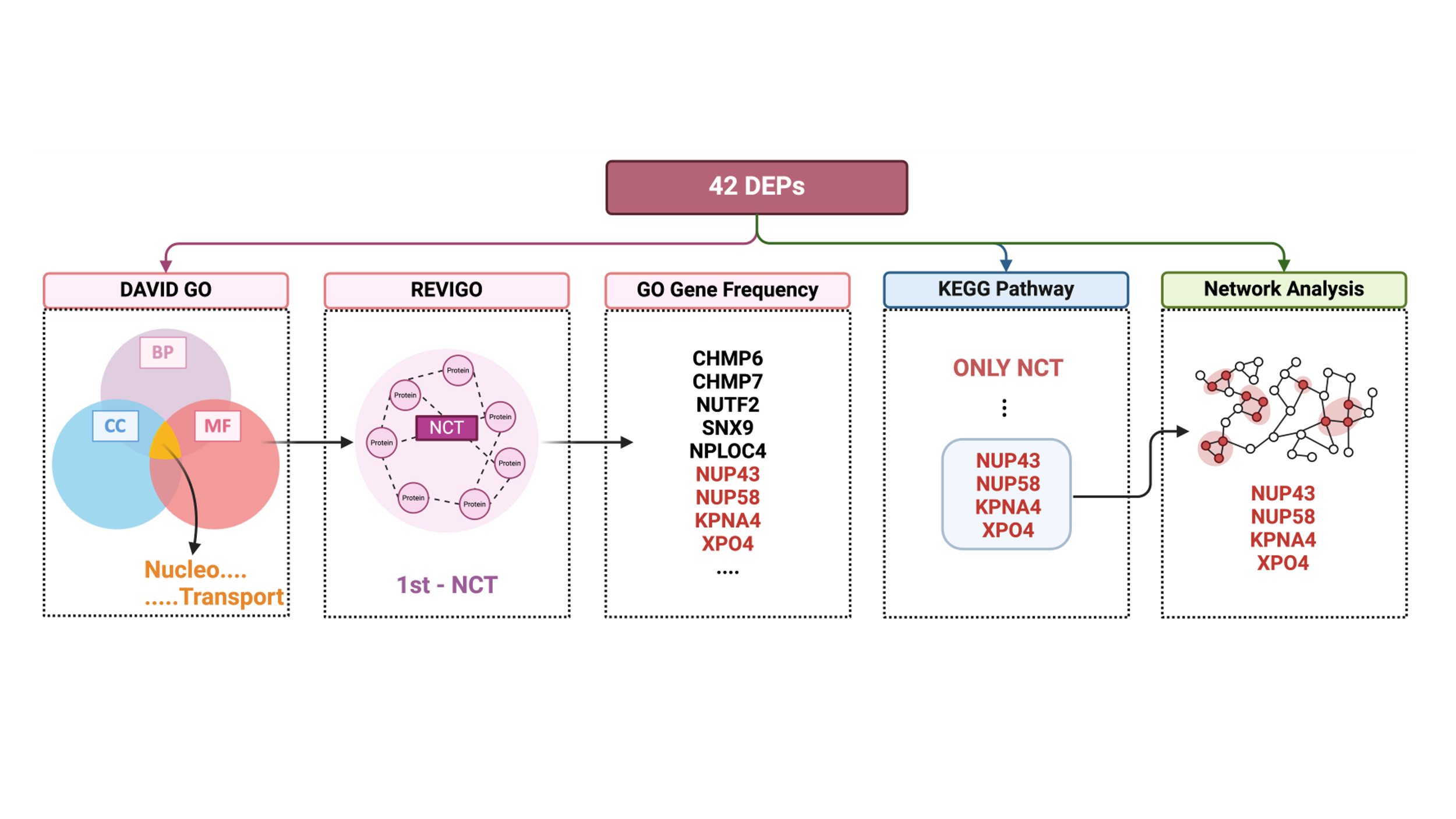
 **Figure S3. Bioinformatic analysis workflow for Cluster 5 of the TMT-based dataset.**

The workflow summarizes the GO enrichment, KEGG pathway analysis, and protein-protein interaction (PPI) network for Cluster 5 DEPs.

**
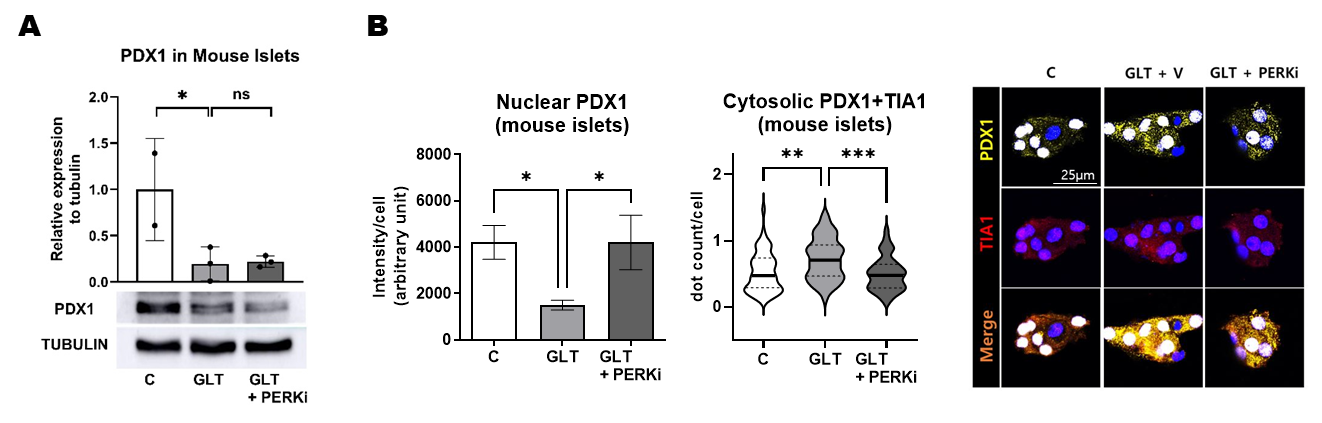
**

**Figure S4. Protein levels and intracellular localization of PDX1 in mouse islets.**

Mouse islets were isolated from adult C57BL/6 mice using ductal injection of collagenase as previously (Moon S et al., Diabetes Metab J. 2024;48(2):231-41.)

(A) Western blot analysis of total PDX1 levels under control, GLT, and GLT+PERKi conditions.

(B) Quantification of nuclear and cytoplasmic PDX1 staining intensity and its co-localization with the stress granule marker TIA1. Representative confocal images show PDX1 (yellow), TIA1 (red), and nuclei counterstained with Hoechst (blue).

Data are expressed as the mean ± standard error of the mean (A-B) and the median and interquartile ranges (B).

*, p < 0.05; **, p < 0.01; ***, p < 0.001 by one-way ANOVA with Tukey's multiple comparisons test.

GLT, glucolipotoxicity; V, vehicle

**Figure S5. Dose determination of HC-5404 in mouse islets.**

Mouse islets were isolated from adult C57BL/6 mice using ductal injection of collagenase as previously (Moon S et al., Diabetes Metab J. 2024;48(2):231-41.) Western blot analysis of phospho-PERK and total PERK levels across various concentrations of HC-5404, a specific PERK inhibitor with a distinct chemical scaffold from GSK2606414 (Calvo V, et al. Bioorg Med Chem Lett. 2021;43:128058). Although IC_50_ of HC-5404 for PERK phosphorylation in cells exposed to tunicamycin was 23 nM (Stokes ME et al, Clin Cancer Res. 2023;29(23):4870-82.), higher concentration was required under physiological concentration in islets. A concentration of 160 nM was selected as its inhibition of phospho-PERK was similar with 40 nM GSK2606414 (GSK).

Data are expressed as mean ± standard error of the mean.

*, p < 0.05; **, p < 0.01; by one-way ANOVA with Tukey's multiple comparisons test.

**
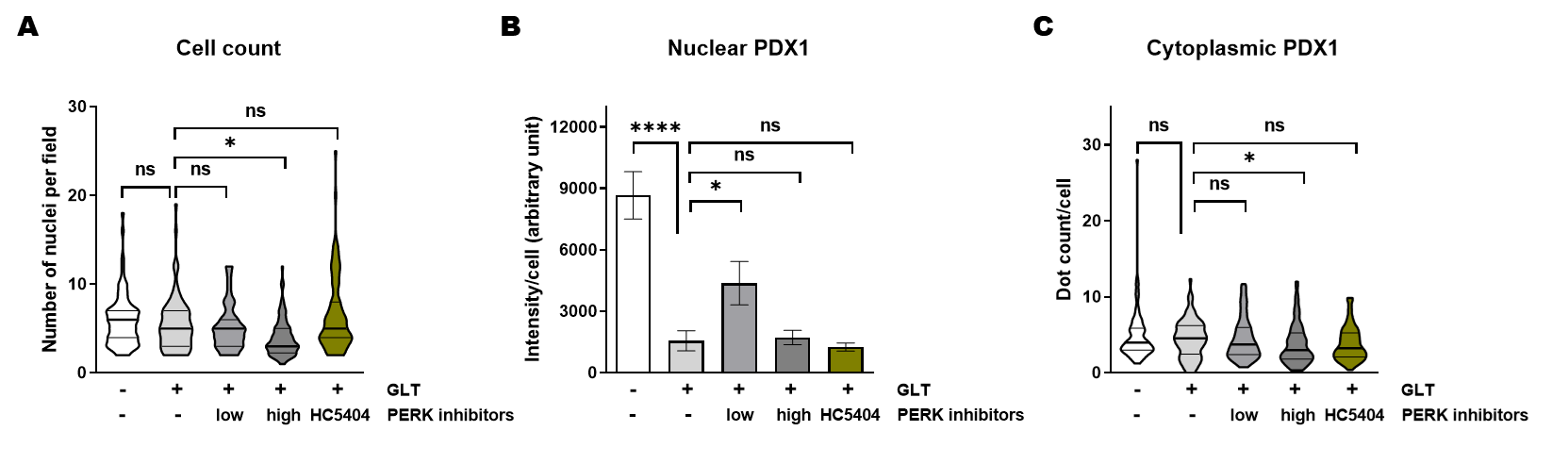
**

**Figure S6. Comparisons of intracellular localization of PDX1 in mouse islets among different doses and classes of PERK inhibitors.**

Mouse islets were isolated from adult C57BL/6 mice using ductal injection of collagenase as previously (Moon S et al., Diabetes Metab J. 2024;48(2):231-41.) Immunocytochemical staining was performed.

(A) Cell viability is assessed by cell counts. High-dose GSK2606414 for 24 h (approximately IC_50_ for EIF2A phosphorylation) significantly decreased cell numbers in GLT conditions, which is compatible with previous findings (Atkins et al, Cancer Res 2013;73:1993–2002; Harding et al. Mol. Cell 7, 1153–1163(2001)).

Quantification of nuclear (B) and cytoplasmic (C) PDX1 staining.

Data are expressed as the median and interquartile ranges (A, C) and mean ± standard error of the mean (B).

*, p < 0.05; ****, p < 0.0001 by one-way ANOVA with Tukey's multiple comparisons test.

GLT, glucolipotoxicity; HC5404, 160-nM HC-5404; high, 400-nM GSK2606414; low, 40-nM GSK2606414


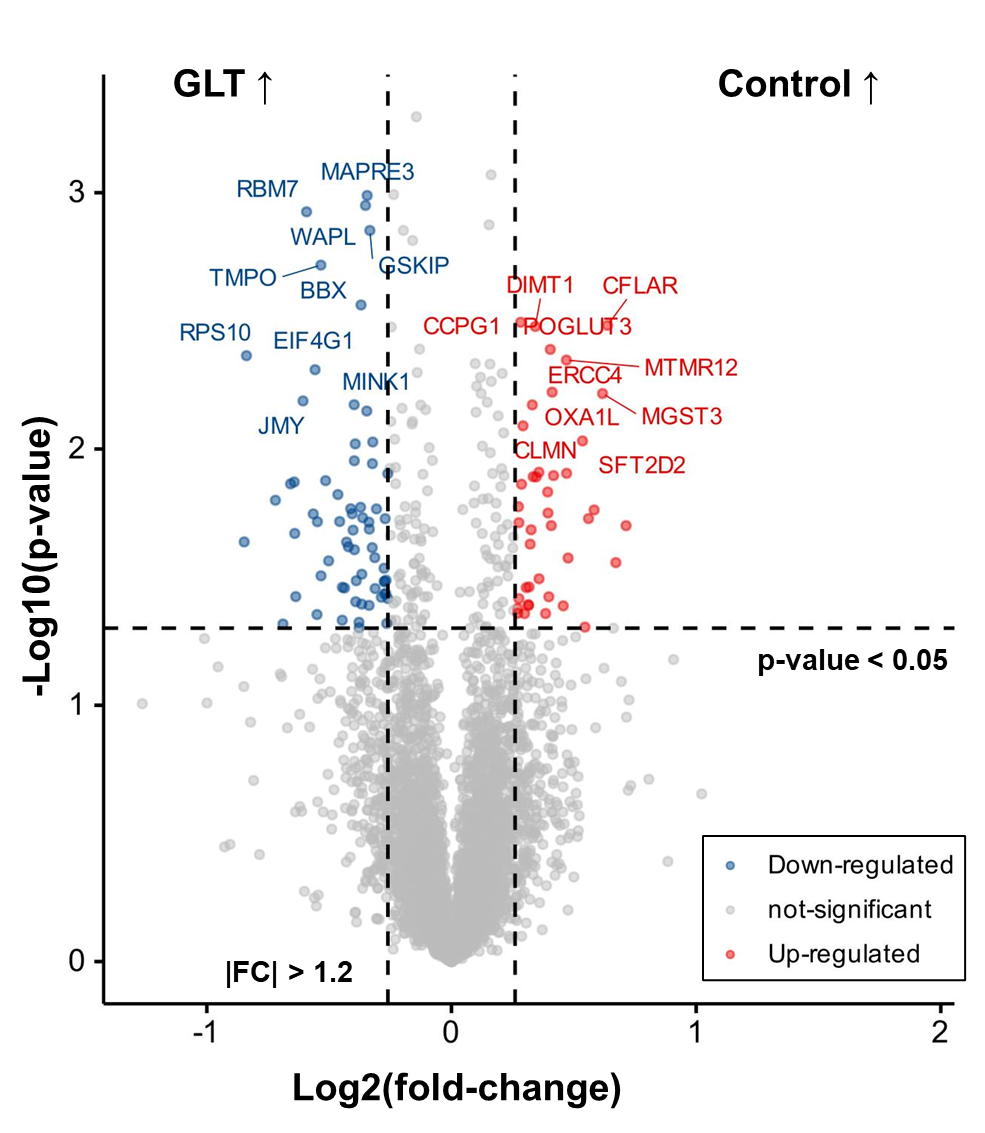


**Figure S7. Volcano plots of DEPs between control and GLT groups in the TMT-based discovery set.**

A total of 106 DEPs were identified based on a fold change > 1.2 and a p-value < 0.05 using Student’s t-test. Blue (65 proteins) and red dots (41 proteins) indicate up- and down-regulated proteins in the GLT groups compared to the control, respectively.
